# Transcriptome and WGCNA reveal hub genes in sugarcane tiller seedlings in response to drought stress

**DOI:** 10.1038/s41598-023-40006-x

**Published:** 2023-08-07

**Authors:** Yuwei Tang, Jiahui Li, Qiqi Song, Qin Cheng, Qinliang Tan, Quanguang Zhou, Zemei Nong, Ping Lv

**Affiliations:** Guangxi Subtropical Crops Research Institute, 22 Yongwu Road, Xingning District, Nanning, 530001 Guangxi Province China

**Keywords:** Biological techniques, Biotechnology, Molecular biology, Plant sciences

## Abstract

Drought stress can severely affect sugarcane growth and yield. The objective of this research was to identify candidate genes in sugarcane tillering seedlings in response to drought stress. We performed a comparative phenotypic, physiological and transcriptomic analysis of tiller seedlings of drought-stressed and well-watered “Guire 2” sugarcane, in a time-course experiment (5 days, 9 days and 15 days). Physiological examination reviewed that SOD, proline, soluble sugars, and soluble proteins accumulated in large amounts in tiller seedlings under different intensities of drought stress, while MDA levels remained at a stable level, indicating that the accumulation of osmoregulatory substances and the enhancement of antioxidant enzyme activities helped to limit further damage caused by drought stress. RNA-seq and weighted gene co-expression network analysis (WGCNA) were performed to identify genes and modules associated with sugarcane tillering seedlings in response to drought stress. Drought stress induced huge down-regulated in gene expression profiles, most of down-regulated genes were mainly associated with photosynthesis, sugar metabolism and fatty acid synthesis. We obtained four gene co-expression modules significantly associated with the physiological changes under drought stress (three modules positively correlated, one module negatively correlated), and found that LSG1-2, ERF1-2, SHKA, TIL, HSP18.1, HSP24.1, HSP16.1 and HSFA6A may play essential regulatory roles as hub genes in increasing SOD, Pro, soluble sugar or soluble protein contents. In addition, one module was found mostly involved in tiller stem diameter, among which members of the BHLH148 were important nodes. These results provide new insights into the mechanisms by which sugarcane tillering seedlings respond to drought stress.

## Introduction

Sugarcane are one of the most important crops in tropical and subtropical regions. It have widespread industrial applications in the production of sugar and ethanol, which also has important economic significance. China is the third largest producer of sugarcane in the world after Brazil and India, and especially, Guangxi produces more than 60% of China’s sugarcane^1^. However, droughts are frequent in Guangxi's main sugarcane producing areas, and limited water resources have exacerbated the impact of arid climate conditions on sugarcane production^2^. Currently, worldwide sugarcane yield (84 ton/ha) is only about 20% of the theoretical potential (381 ton/ha)^3^, and thus, the yield of sugarcane still leave much room for improvement, and this process is known as tillering. sugarcane yield is largely determined by tillering, which is mediated by environmental and genetic factors.

Sugarcane can be harvested for many years and the plant is mainly composed of stalks, which are derived from tillers. The underground part of the sugarcane stem sprouts new seedlings, and then healthy seedlings further develop into effective stalk. Drought is one of the major natural disaters for agriculture worldwide, and severely hampers sugarcane growth at the seedling tillering stage. Therefore, it is not only necessary to select and breed varieties with strong tillering ability, but also drought-tolerant varieties in sugarcane breeding.

Osmotic regulation and antioxidant defense are important strategies in the resistance of plants to drought stress damage. Osmoregulation relies on the accumulation of osmoprotectants or osmoregulators, such as soluble proteins, soluble sugars and proline^4^. Antioxidant enzymes mainly include superoxide-dismutase (SOD), and peroxidase (POD), which are activated to detoxify ROS under drought stress^5^. Plant hormones such as auxin (IAA), abscisic acid (ABA), gibberellin (GA), cytokinins (CKs) and strigolactons (SLs) play a crucial role in crop growth and yield under drought stress^6^. For example, ABA can regulate stomata to reduce water lost significantly^7^, SLs can reduce H_2_O_2_, malondialdehyde (MDA), and improved water content and membrane stability in the drought-exposed wheat and *Vitis vinifera* ^8^. The reduce of GA activity slow down the growth of plant that can effectively alleviate the damage caused by drought stress^9^. IAA improved drought stress tolerance connected with delayed leaf senescence^10^.

Over the years, transcriptomics technology has been widely applied to investigate the molecular mechanism of plants in response to drought stress. As is well known, weighted gene co-expression network analysis (WGCNA) is one such tool to discover correlation patterns among genes and identify the relationship between co-expressed modules and trait. In recent years, WGCNA has been performed to identify hub genes in response to abiotic stress in crops, such as rice^11^, wheat^12^ and maize^13^. Here, we conducted a comprehensive analysis between gene expression patterns and physiological indicators of drought response in sugarcane tillering seedlings by WGCNA, identified candidate genes associated with response to drought.

## Result

### Phenotypic changes of sugarcane tillering seedlings under drought stress

**Figure 1:**
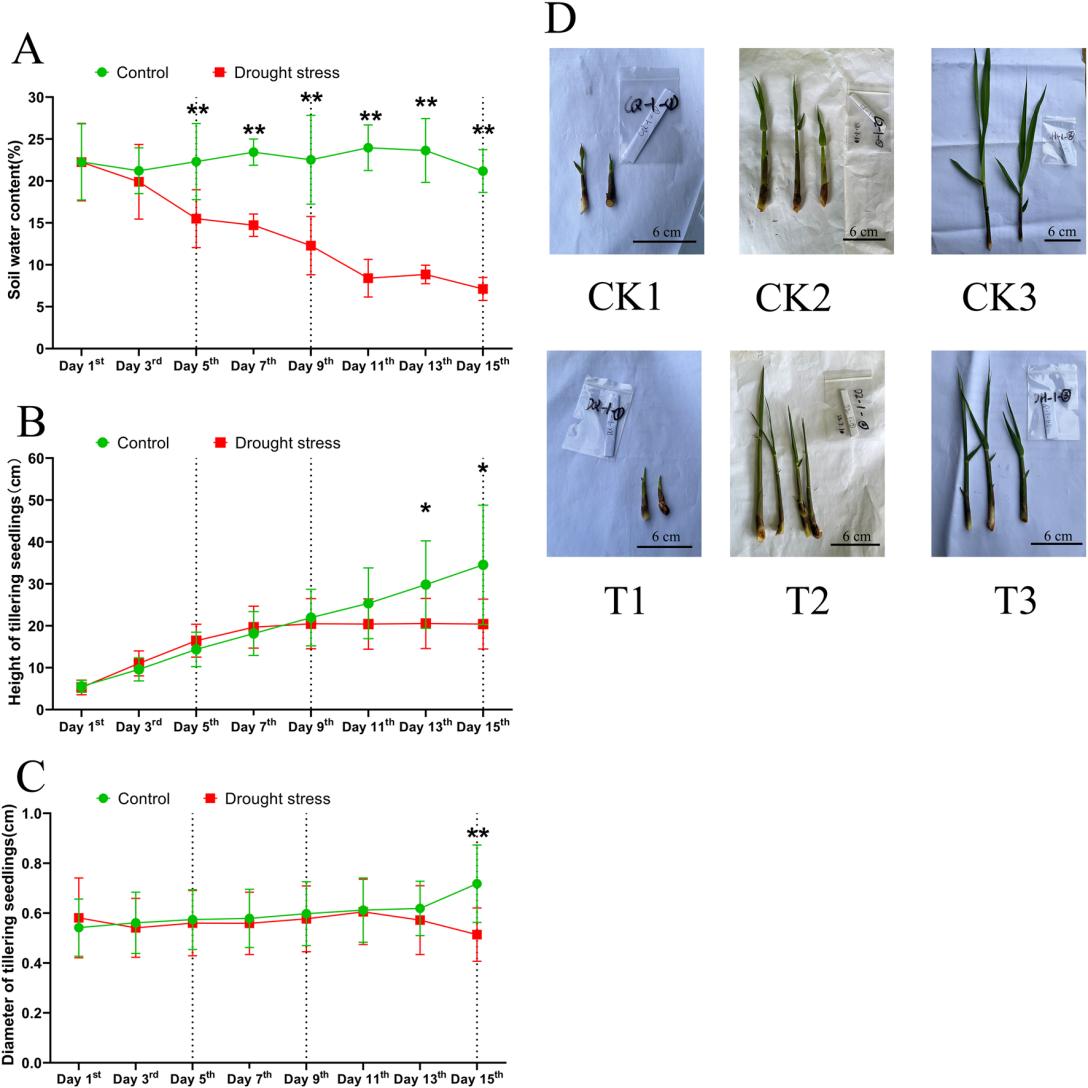
Effect of drought stress on sugarcane. * and ** indicate the significant difference at 5% level and 1% level, respectively (independent t-test). (**A**) Soil water content. (**B**) Height of tillering seedlings. (**C**) Diameter of tillering seedlings. (**D**) Phenotypic changes of tiller seedlings in response to the drought-treated and well-watered control at three time points (5, 9 and 15 days).

“Guire 2” were planted in plastic pots and treated with drought at the tiller formation stage. The experiment applied drought stress by stop watering. The changes of soil water content (SWC) in the control group and drought treatment groups were shown in Fig. 1A. After 15 days of drought stress, the SWC in the treatment group decreased significantly from 22.25 to 7.12%. After 15 days of well watering, the SWC in the control group was maintained at about 21–24%. From 5 days onwards, SWC was significantly different between both groups, consistently.

In the first 11 days of drought stress, experience group and watering control group did not have significant differences in tiller height. However, On the 13th and 15th day of drought stress, the height of tillering seedlings in the experimental group was significantly lower than that of the control group (P < 0.05). On the 15th day, the stem diameter was significantly lower than that of the control group (P < 0.01) (Fig. 1B,C).

The phenotypes of tiller seedlings in the treatment and control groups were almost identical in the pre-drought (5th day) and mid-drought periods (9th day). And yet, the growth of “Guire 2” tiller seedlings becomes sluggish and tiller leaves begin to curl in late-drought (15th day) (Fig. 1D).

Base on the SWC and tiller phenotypic changes, we selected sugarcane that was drought stressed on day 5 (T1), day 9 (T2) and day 15 (T3) as the experimental groups, and sugarcane watered normally on day 5 (CK1), day 9 (CK2) and day 15 (CK3) as the control groups. The above six sets of tiller seedlings were further used for physiological assays and transcriptome sequencing.

### Physiological changes of “Guire 2” tiller seedlings under drought stress

In order to study the physiological responses of sugarcane 'Guire 2' tiller seedlings to drought stress, the physiological indices such as superoxide dismutase (SOD), peroxide dismutase (POD), malondialdehyde (MDA), indoleacetic acid (IAA) and zeatin (ZT) were evaluated at the tiller formation stage.

**Figure 2:**
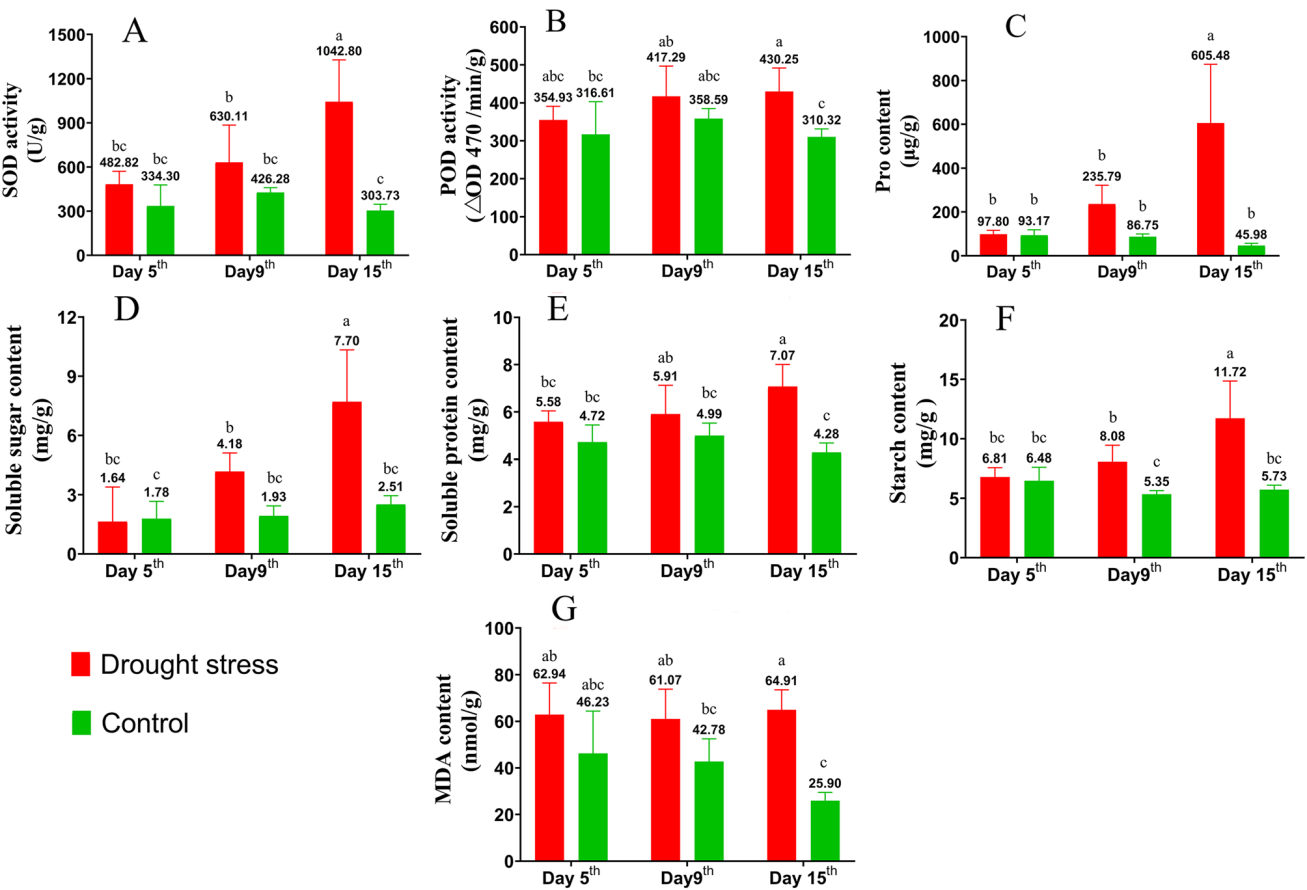
Physiological changes of “Guire 2” tiller seedlings under drought stress (treated group) or well watering treatment (control group)**.** Different letters (a–c) represent significant differences (LSD, *P* < 0.05). (**A**) Superoxide dismutase (SOD) activity. (**B**) Peroxide dismutase (POD) activity. (**C**) Proline (Pro) content. (**D**) Soluble sugar content (SS). (**E**) Soluble protein content (SP). (**F**) Starch content. (**G**) Malondialdehyde (MDA) content.

In terms of oxidative stress, drought stress results in the production of reactive oxygenspecies (ROS), and excessive ROS would lead to oxidativestress, inhibit plant growth, and even cause cell death. Antioxidant enzymes such as SOD and POD form a defense system and play critical roles in removing the excessive ROS induced by drought stress. In our researcher, SOD activity increased from 482.82 U/g to 1042.90 U/g with the in creasing of drought treatment time. Compared with the treated group, the SOD activity of the control group was always maintained at a lower level (Fig. 2A). Results of POD activity showed that there was no significant difference between the drought stress and control on day 5 and day 9. Neverless, the drought stress group was significantly (p < 0.05) higher than the control group at 15 days of drought stress (Fig. 2B). Osmoregulatory substances play an equally important role in the response of plants to drought stress. In terms of osmoregulation, the result of proline (Pro) content showed no significant difference between the treatment and control groups on day 5 day 9 of drought stress. But, the Pro content of the control group increased significantly on the 15 day of drought stress (Fig. 2C). Soluble sugar (SS) and soluble protein (SP) contents, as well as starch content, remained consistent throughout the drought treatment and increased as drought stress increased. On day 5, there was no significant difference between the SS, SP and starch of treated and control groups. However, on the 15th day, in the treated group, SS, SP and starch content were significantly (P < 0.05) raised when compared with the control group (Fig. 2D–F). MDA is an important signal to evaluate plant cell damage. Results of MDA content showed that the drought-stressed group was consistently higher than the control group. But surprisingly, the MDA content did not increase with the increase of drought stress time and always maintained at a stable level (Fig. 2G). The data changes of MDA content imply that drought stress did not cause greater cellular damage to tiller seedlings within the experimental range.

Drought stress leads to changes in endogenous hormones from sugarcane tiller seedlings at each time point, as shown in Supplementary Fig. 1. A significant (P < 0.05) increase in ABA content was observed in all drought stress treatments over the control (Supplementary Fig. 1A). Drought stress treatment significantly (P < 0.05) increased GA_3_ levels only on day 5 compared with the control, but in response to the increase of drought stress, GA_3_ content first decreases and then increases (Supplementary Fig. 1B). Similarly, the SLs content of the drought stress group also showed the same trend (Supplementary Fig. 1E). The IAA content exhibited a greater increase at 15 days than 5 and 9 days of drought stress. In addition, on day 9, the IAA content in the control group was significantly (P < 0.05) higher than that in the drought-stressed group (Supplementary Fig. 1C). Result of ZT content displayed that the differences between drought-stressed group and control group were significant at day 5 and 9, whereas on day 15, there were no longer significant differences between drought-stressed group and control group (Supplementary Fig. 1D).

### RNA sequencing analysis of “Guire 2” tiller seedlings under drought stress

To establish the key genes of sugarcane tiller seedlings in response to drought, the treated tiller seedlings of “Guire 2” were sequenced. A total of approximately 151.64 million raw reads were generated from the 18 (6 samples × 3 replications) cDNA libraries. After deleting 0.03 ~ 0.04% of adapter sequences, and filtering 0.31 ~ 0.41% of low-quality reads and 0.00% of n-containing reads, a total of approximately 151.01 million high-quality clean reads were finally confirmed (Supplementary Table 1), with an average Q20 and Q30 value of 97.82% and 93.84%, respectively (Supplementary Table 2). The mapping rate to the reference genome ranges from 82.91% to 88.31% (Supplementary Table 3). These results showed that the transcriptome sequencing quality was sufficient for further analyses.

**Figure 3:**
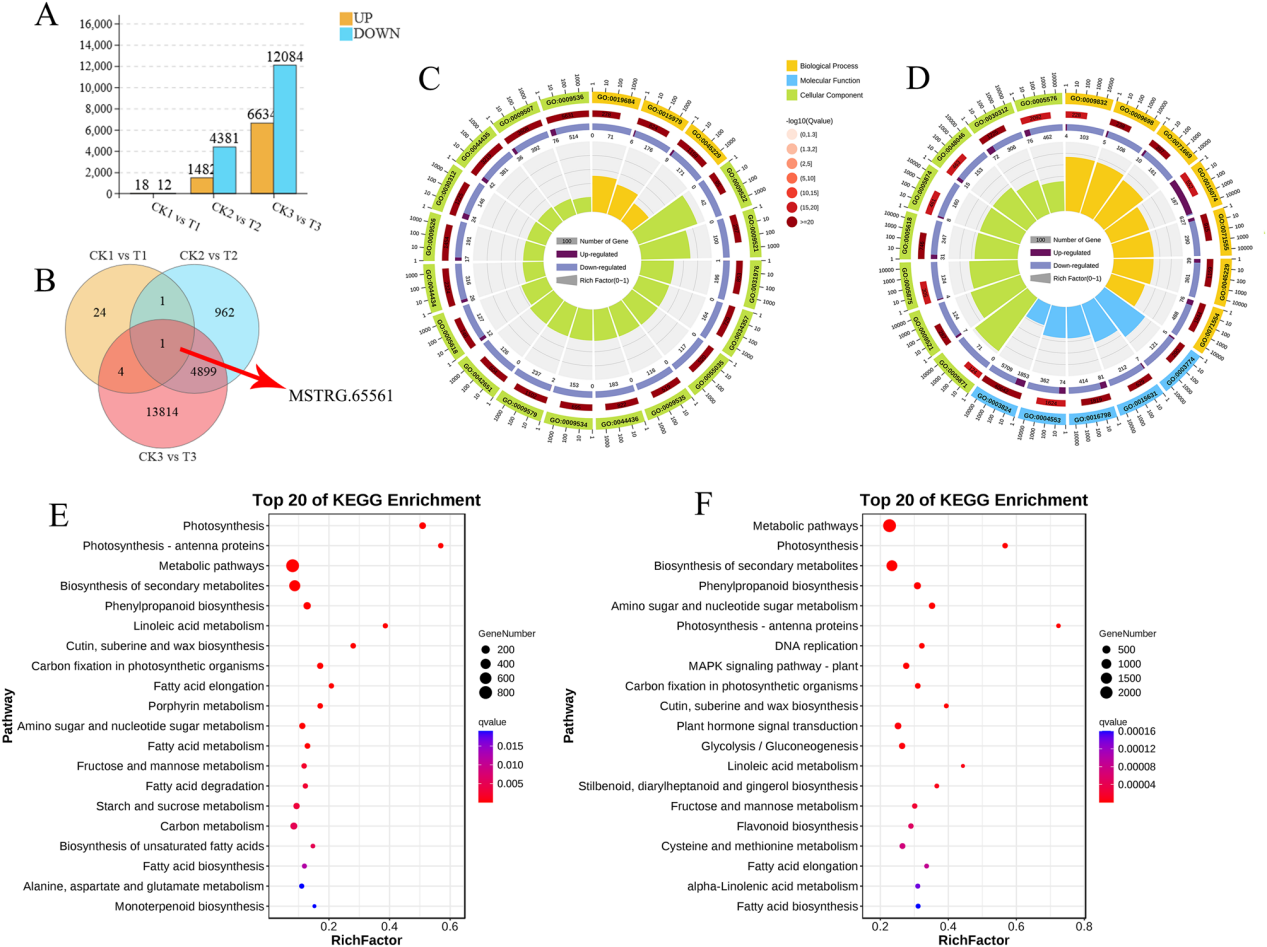
Difference analysis of gene expression by pairwise comparisons. CK1 vs T1: comparison between 5 days of drought and 5 days of well-watered condition; CK2 vs T2: comparison between 9 days of drought and 9days of well-watered condition; CK3 vs T3: comparison between 15 days of drought and 15 days of well-watered condition. (**A**) The number of DEGs induced under drought stress. (**B**) Venn diagram of DEGs at the three-points under drought stress. (**C**) GO enrichment circle diagram of DEGs in CK2 vs T2. (**D**) GO enrichment circle diagram of DEGs in CK3 vs T3. First circle: qvalue value of the top 20 GO term. Second circle: the number of all genes in this GO term and the Q value. Third circle: up- and down-regulated differential gene ratio bars, dark purple represents up-regulated DEGs, light purple represents down-regulated DEGs. Fourth circle: RichFactor values for each GO term, each grid represents 0.1 in background grid lines. (**E**) KEGG pathway analysis of DEGs in CK2 vs T2. (**F**) KEGG pathway analysis of DEGs in CK3 vs T3.

Normally, a stringent threshold absolute log_2_ FC ≥ 2 and FDR < 0.05 was used to screen out DEGs, GO enrichment and KEGG pathway analysis were applied to assay these DEGs to further understand their biological function. After 5 days of drought treatment, 18 up-regulated DEGs and 12 down-regulated DEGs were identified. The DEGs after 9 days of drought treatment, 1482 were up-regulated and 4381 were down-regulated. After 15 days of continuous drought treatment, the number of DEGs was the largest in the three treatment with 6634 up-regulated genes and 12,084 down-regulated genes (Fig. 3A). On the whole, the number of down-regulated DEGs is higher than up-regulated DEGs, except for drought stress for 5 days. Together, the results revealed that the number of induced DEGs greatly increased with the continuation of drought stress time. Venn diagram shows one common DEG (MSTRG.65561) was consistently involved in CK1 vs T1, CK2 vs T2, and CK3 vs T3 (Fig. 3B), and the expression of this gene increased substantially with the enhancement of drought stress. However, MSTRG.65561 could not be annotated according to the KEGG and GO databases, and consequently its biological function could not be determined (Supplementary table 4).

In order to understand the function of these DEGs, GO enrichment analysis was performed for DEGs. GO enrichment analysis of CK1 vs T1 showed that the qvalue of GO enrichment was greater than 0.01, which was not analytically significant (Supplementary table 5). In CK2 and T2, DEGs were mainly enriched in 3 biological processes, include “photosynthesis, light reaction ”(GO:0019684) , “photosynthesis” (GO:0015979) and “external encapsulating structure organization” (GO:0045229). Especially, the rich factor of GO:0019684 and GO:0015979 associated with photosynthesis were higher. Among the top 20 significantly enriched GO terms, 17 GO terms belong to cellular composition, they were “photosystem I” (GO:0009522), “photosystem” (GO:0009521), “plastid thylakoid” (GO:0031976), “photosynthetic membrane” (GO:0034357), “plastid thylakoid membrane” (GO:0055035), “chloroplast thylakoid membrane”(GO:0009535), “thylakoid part” (GO: 0044436), “chloroplast thylakoid” (GO:0009534), “thylakoid” (GO:0009579), “thylakoid membrane” (GO:0042651), “cell wall” (GO:0005618), “chloroplast part” (GO:0044434), “plastid envelope” (GO:0009526), “external encapsulation structure” (GO:0030312), “plastid part” (GO: 0044435), “chloroplast” (GO:0009507) and “plastid” (GO:0009536), respectively (Fig. 3C and supplementary table 6). Photosynthesis was seriously impacted in “Guire 2” tiller seedlings at 9 days of drought stress, as evidenced by GO enrichment analysis of CK2 vs T2.

The DEGs of CK3 vs T3 goup were subjected to a GO analysis comprising 7 biological processes, 8 cellular components, and 5 molecular functions among the top 20 significantly enriched GO terms (Fig. 3D and supplementary table 6). GO terms of molecular functions were increased, which were “catalytic activity” (GO:0003824), “hydrolase activity, acting on glycosyl” (GO:0016798), “tubulin binding” (GO:0015631), “motor activity” (GO:0003774), “hydrolase activity, hydrolyzing O-glycosyl compounds” (GO:0004553), respectively. As shown in Fig. 3D, most genes were down-regulated in GO terms of molecular function, thus we speculate that the hydrolase activity of tiller seedlings was reduced at this time due to drought stress. These GO enrichments of cellular components include “cell wall” (GO:0005618), “external encapsulating structure” (GO:0030312), “photosystem” (GO:0009521), “microtubule associated complex” (GO:0005875), “kinesin complex” (GO:0005871), “microtubule” (GO:0005874), “apoplast” (GO:0048046), “extracellular region” (GO:0005576). GO term for biological processes include: “external encapsulating structure organization” (GO:0045229), “cell wall organization or biogenesis” (GO:0071554), “cell wall organization” (GO:0071555), “plant-type cell wall organization or biogenesis” (GO:0071669), “phenylpropanoid metabolic process” (GO:0009698), “plant-type cell wall biogenesis occurrence” (GO:0009832), “DNA integration” (GO:0015074). We found that a number of significantly enriched GO items were associated with the development of cell wall tissue, and in particular, most of DEGs were down-regulated (Fig. 3D). The results indicate possibly that the cell wall development of tiller seedlings was negatively affected, thus corroborated our previous results that the height and stem diameter traits of the "Guire 2" treatment group were significantly lower than those of the control group (Fig. 1A,B).

The results of the KEGG enrichment analysis are shown in Fig. 3 E and F with the first 20 top-ranking pathways indicated by the smallest significant qvalues. KEGG pathway enrichment analysis indicated that DEGs involved in photosynthesis, sugar metabolism and fatty acid metabolism pathways in both the mid (CK2 vs T2) and late drought periods (CK3 vs T3). For example, “Photosynthesis” (ko00195), “Photosynthesis—antenna proteins” (ko00196), “Carbon fixation in photosynthetic organisms” (ko00710) are related to photosynthesis. “Fructose and mannose metabolism” (ko01212), “Starch and sucrose metabolism” (ko00500) are related to sugar metabolism. “Carbon fixation in photosynthetic organisms” (ko00710) 、”Fatty acid elongation” (ko00062) are related to fatty acid metabolism.

### WGCNA of “Guire 2” tiller seedlings under drought stress

The weighted gene co-expression network analysis (WGCNA) was used to analyze the connection between genes and physiological traits, discovering the hub genes associated with physiological and biological traits. Genes with a max *FPKM* of < 15were filtered out, and the 16,415 selected genes were assigned to 20 merged co-expression module with various colors (Supplementary Fig. 2). The weight value indicates the correlation of gene relationship pairs in the module, one relationship pair for every two genes. We selected the top 100 relationship pairs in terms of weight to draw the network regulatory map, and then selected the five genes with the highest degree of connectivity as the hub genes from them. It is commonly recognized that hub genes form the backbone of the network and tend to be critical in particular physiological events^14^.

**Figure 4:**
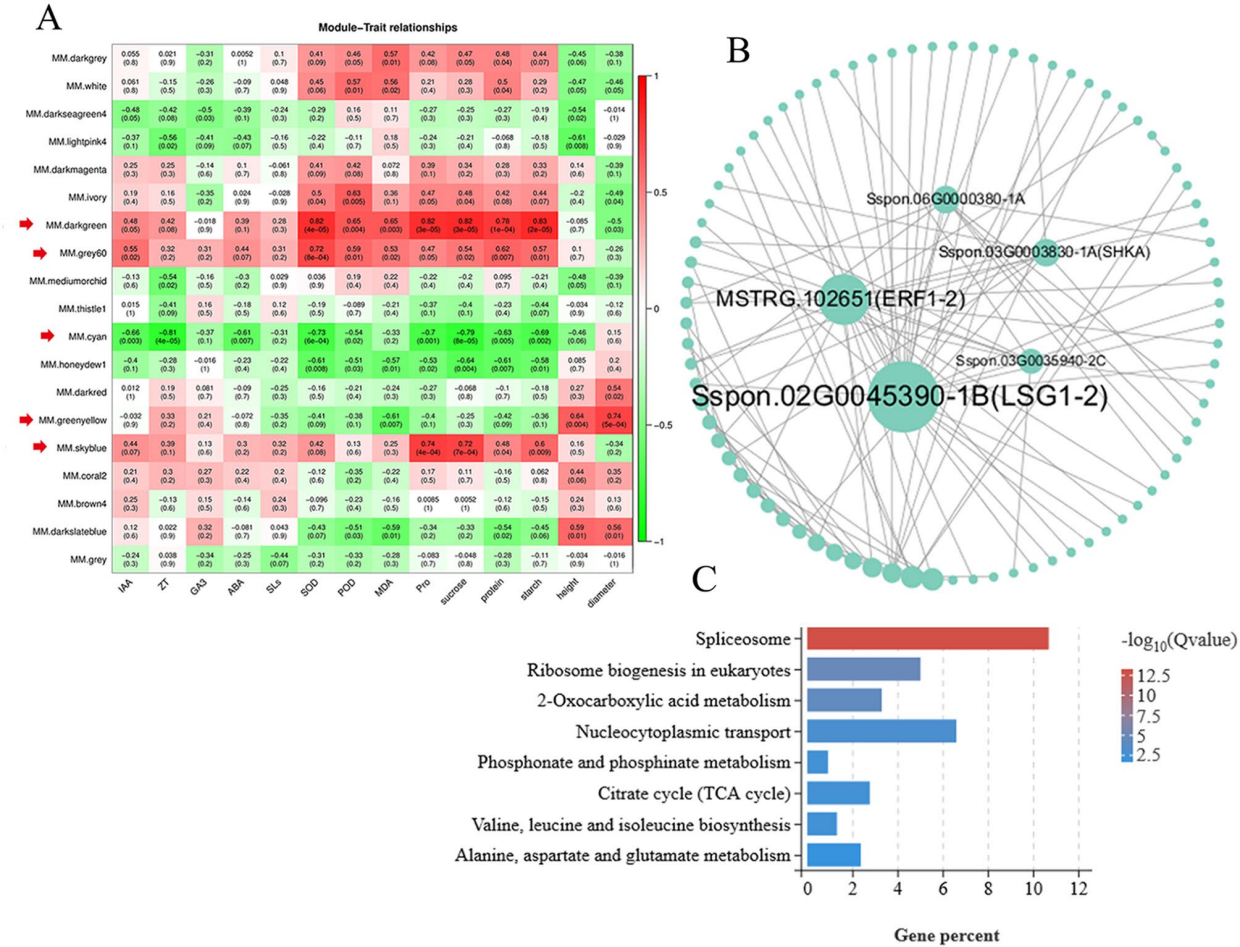
WGCNA module identification and correlation analysis. (**A**) the correlation of the identified modules with the physiological or biological traits under drought stress. Red and green color notes positive and negative correlation with gene expression, respectively. (**B**) Gene interaction network analysis in the darkgreen modules, the bigger the node, the greater the number of connections it has. The center area of the circle were candidate hub genes. (**C**) KEGG pathway enrichment analysis of the darkgreen modules.

As showed in Fig. 4A, we successfully identified five modules significantly (r >|0.7|) associated with physiological or biological traits of “Guire 2” tiller seedlings under drought stress. Based on the analysis results of the WGCNA co-expression module, the coexpression network of the darkgreen, grey60, skyblue, cyan and greenyellow modules were further analyzed. Additionally, the darkgreen module was positively correlated with SOD (*r* = 0.82, *p* = 4e−05) , Pro (*r* = 0.82, *p* = 3e−05) , sucrose (*r* = 0.82, *p* = 3e−05) , protein (*r* = 0.78, *p* = 1e−04) and starch content (*r* = 0.83, *p* = 2e−05) under drought stress. The grey60 module was positively correlated with SOD content (*r* = 0.72, *p* = 8e−04) and also showed a good positive correlation with other physiological indicators. The skyblue module had a significant positive correlated with Pro (*r* = 0.74, *p* = 4e−04) and sucrose content (*r* = 0.72, *p* = 7e−04), while the cyan module was negatively correlated with ZT (*r* = −0.88, *p* = 0.003), SOD (*r* = −0.73, *p* = 6e−04), Pro (*r* = −0.7, *p* = 0.001) and sucrose (*r* = −0.79, *p* = 8e−05) content under drought stress. Particularly, the greenyellow module had a significant positive correlated with tiller stem diameter (*r* = 0.74, *p* = 5e−04).

We found that the darkgreen, grey60 and skyblue module were positively correlated with the physiological of "Guire 2" tiller seedlings (Fig. 4A). The correlation network of the darkgreen module is shown in Fig. 4B, Sspon.02G0045390-1B (LSG1-2), MSTRG.102651 (ERF1-2), Sspon.03G0003830-1A, Sspon.06G0000380-1A (SHKA), Sspon.03G0035940-2C were identified as candidate hub genes for this module(Supplementary table 8). In darkgreen module, eight significant pathways (qvalue < 0.05) are identified, and “Spliceosome” (ko03040) pathway was the most significant enrichment of genes (Fig. 4C).

**Figure 5:**
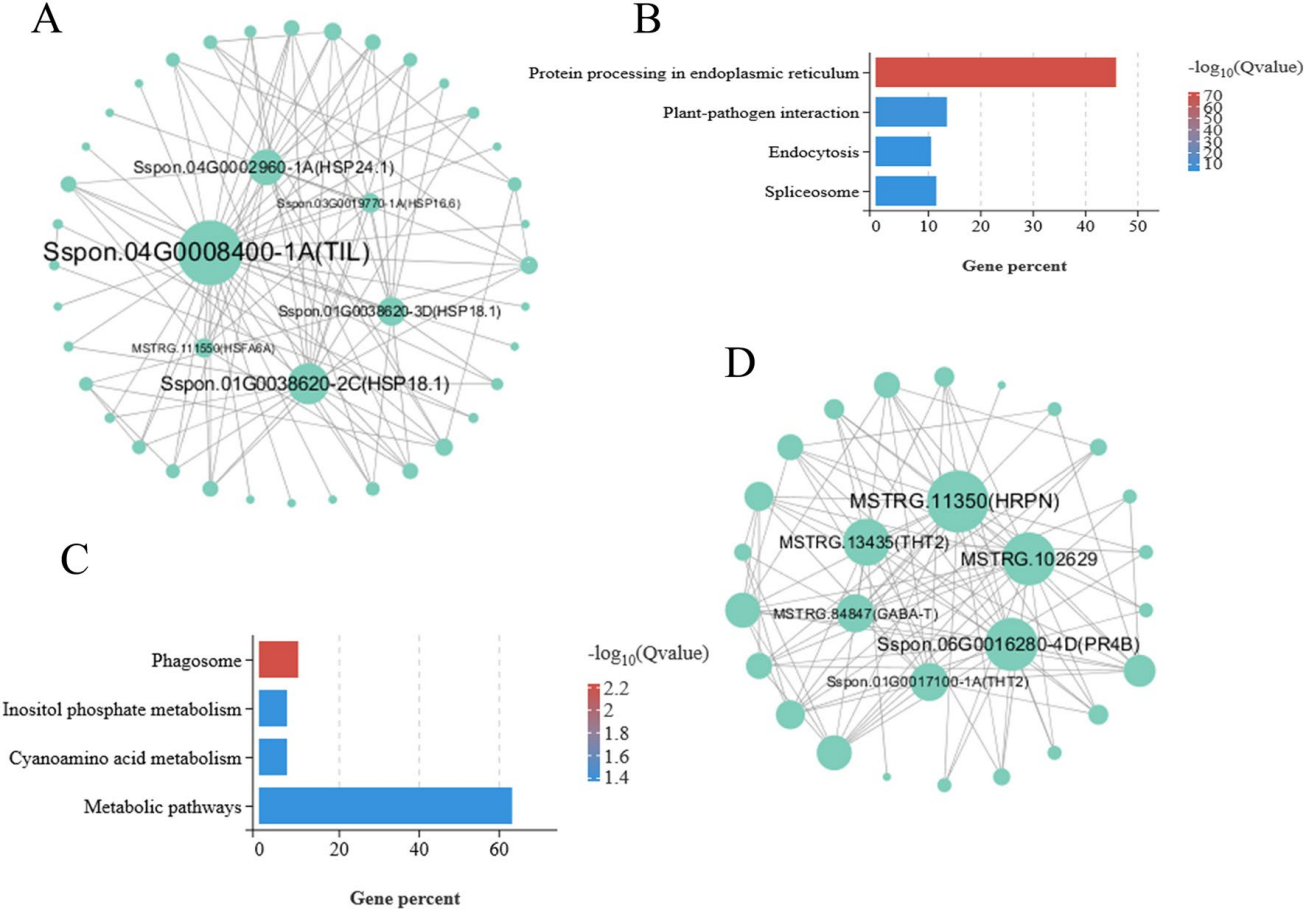
(**A**) Gene interaction network analysis in the grey60 module. (**B**) KEGG pathway enrichment analysis of the grey60 module. (**C**) KEGG pathway enrichment analysis of the skyblue module. (**D**) Gene interaction network analysis in the skyblue module.

The correlation network of the grey60 module is shown in Fig. 5A, Sspon.04G0008400-1A (TIL), Sspon.01G0038620-2C (HSP18.1), Sspon.04G0002960-1A (HSP24.1), Sspon.01G0038620-3D (HSP 18.1), Sspon.03G0019770-1A (HSP16.1), MSTRG.111550 (HSFA6A) were regarded as module candidate hub genes(Supplementary table 8). Among these candidate hub genes, four genes belong to small molecule heat shock proteins (sHSPs), indicating that sHSP regulatory network may play a major role in SOD regulatory of drought stress. In grey60 module, KEGG pathway analysis showed that “Protein processing in endoplasmic reticulum” (ko04141), “Plant-pathogen interaction” (ko04626), “Endocytosis” (ko04144) and “Spliceosome” (ko03040) were most significantly (qvalue < 0.05) enriched by these genes (Fig. 5B).

As showed in Fig. 5C, the skyblue module could be signifiantly (qvalue < 0.05) enriched in “Phagosome” (ko04145), “Inositol phosphate metabolism” (ko00562), “Cyanoamino acid metabolism” (ko00460) and “Metabolic pathways” (ko01100). The correlation network of the skyblue module is shown in Fig. 5D, MSTRG.11350, MSTRG.102629, Sspon.06G0016280-4D, MSTRG.13435, Sspon.01G0017100-1A and MSTRG.84847 were identified as candidate hub genes for this module(Supplementary table 8).

**Figure 6:**
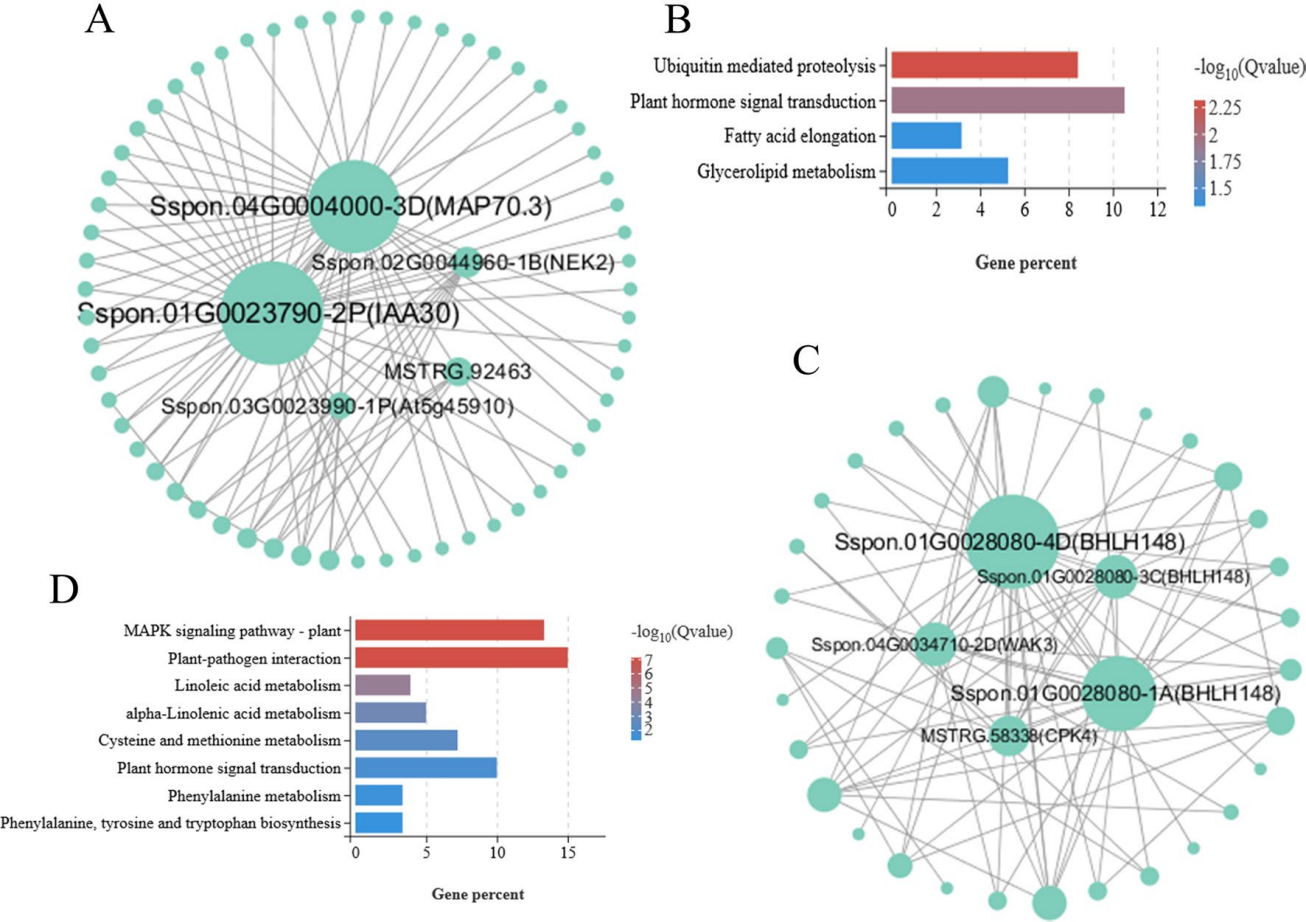
(**A**) Gene interaction network analysis in the cyan module. (**B**) KEGG pathway enrichment analysis of the cyan module. (**C**) Gene interaction network analysis in the greenyellow module. (**D**) KEGG pathway enrichment analysis of the greenyellow module.

The cyan module negatively correlated to physiological traits of tiller seedlings, and the genes in this module may have a suppressive effect on the physiological regulation of tiller seedlings. The correlation network shows that Sspon.01G0023790-2P (IAA30), Sspon.04G0004000-3D (MAP70.3), Sspon.02G0044960-1B (NEK2), MSTRG.92463 (CLASP) and Sspon.03G0023990-1P (At5g45910) were identified as candidate hub genes for the cyan module (Fig. 6A and Supplementary table 8). In additional, For KEGG pathway enrichment analysis as showed in Fig. 6B, the result showed that these genes were significantly (qvalue < 0.05) enriched in “Ubiquitin mediated proteolysis” (ko04120), “Plant hormone signal transduction” (ko04075), “Fatty acid elongation” (ko00062), “Glycerolipid metabolism” (ko00561).

Greenyellow module was positively correlated with stem diameter development of tiller seedlings. The correlation network of the skyblue module is shown in Fig. 6C, Sspon.01G0028080-4D (BHLH148), Sspon.01G0028080-1A (BHLH148), Sspon.04G0034710-2D (WAK3), Sspon.01G0028080-3C (BHLH148) and MSTRG.58338 (CPK4) were identified as candidate hub genes for this module(Supplementary table 8). Notably, members of the BHLH148 were important nodes with abundant connection points in the co-regulatory network, indicating that BHLH148 may be crucial to tiller seedlings development under drought stress. KEGG enrichment analysis for greenyellow module genes as showed in Fig. 6D, the most significantly (qvalue < 0.5) enriched pathways were “MAPK signaling pathway—plant” (ko04016), “Plant-pathogen interaction” (ko04626), “Linoleic acid metabolism” (ko00591), “alpha-Linolenic acid metabolism” (ko00592), “Cysteine and methionine metabolism” (ko00270), “Plant hormone signal transduction” (ko04075), “Phenylalanine metabolism” (ko00360) and “Phenylalanine, tyrosine and tryptophan biosynthesis” (ko00400).

### Validation of candidate hub gene expression changes by qRT*-*PCR

**Figure 7:**
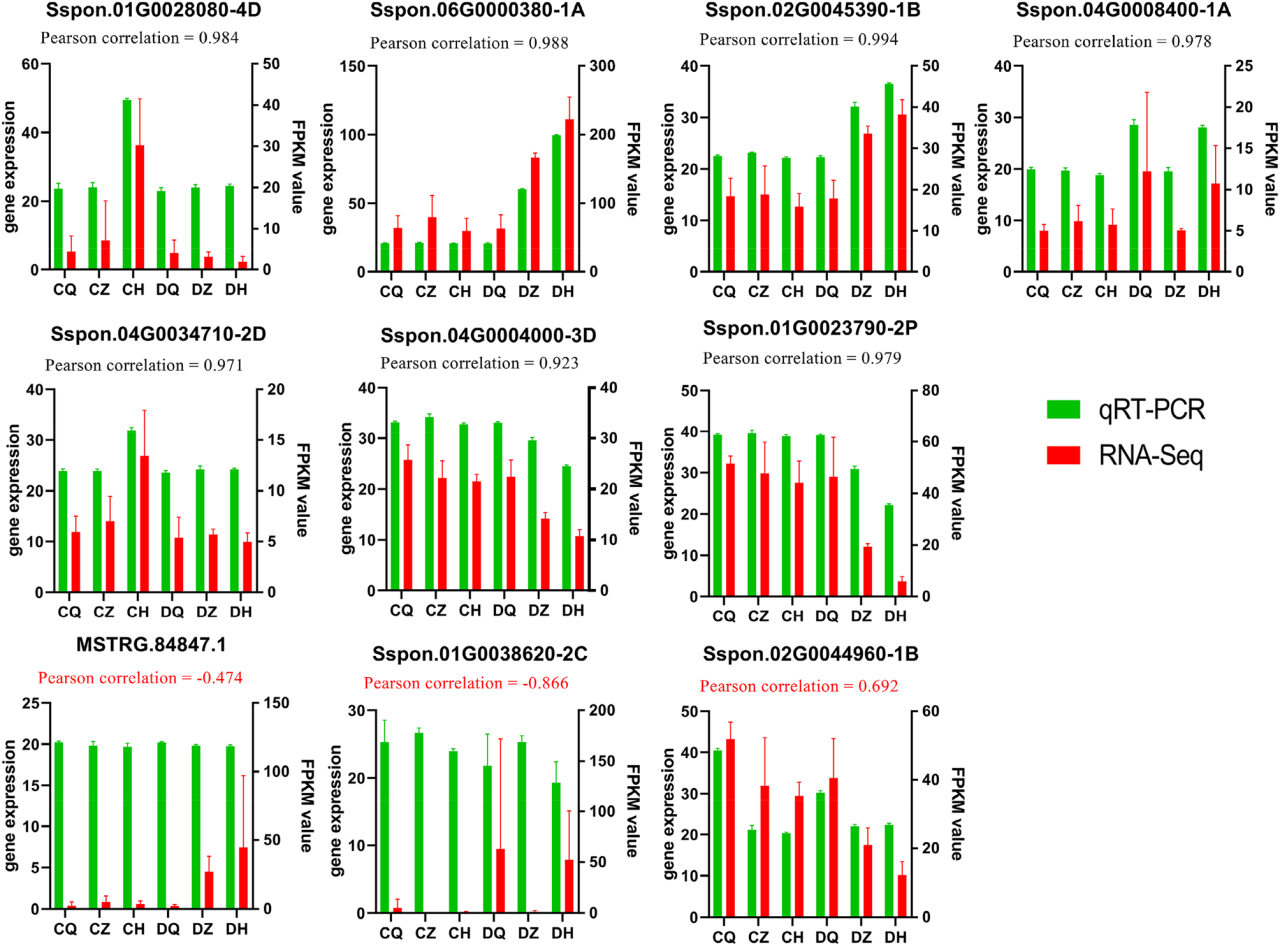
qRT-PCR validation of 10 candidate hub genes. The column chart shows the FPKM values in RNA-seq data. Correlation analysis was performed with Pearsons correlation test.

To verify the accuracy and reproducibility of the transcriptome analysis, 10 candidate hub genes were randomly selected to analysis the transcript abundance by quantitative real–time PCR (qRT-PCR). The results showed that most of genes the expression profiles detected by qRT-PCR were positively correlated (pearson correlation > 0.9) with the RNA-Seq results, only 3 candida te hub genes was inconsistent with the RNA-Seq results (Fig. 7). Overall, the reliability of the RNA-seq data was confirmed by the consistency between the qRT-PCR results and RNA-seq analyses.

## Discussion

Removing reactive oxygen species (ROS) and regulating osmotic pressure are important metabolic strategy in regulation of plant responses to drought stress. Under normal condition, ROS in plant cells were in a dynamic equilibrium, and maintained at low levels by antioxidative enzyme, thus exert no lasting damage to plant cells^15^. Water shortage cause overproduction of ROS in the plant, thereafter leading to disrupt redox homeostasis, which is harmful to plant growth and development^16^. To alleviate this deleterious effects, plants produce a lots of ROS-scavenging enzymes, such as SOD and POD, which could ensure their survival under drought stress^17^. In a previous study, Rayyan Khan et al. found that drought-resistant tobacco mitigated the oxidative damage by increasing the antioxidant enzyme activities and elevated the content of antioxidant substances^18^. The same conclusion was obtained by Chengke Luo et al*.* in a drought stress study in rice, rice sprayed with melatonin (drought regulator) could accumulate more SOD and POD to mitigate the damage caused by drought stress to seedlings^19^. Osmoregulation is another important defense strategy under drought stress, which effectively reduce the water potential of plant cells, prevent cell dehydration and ensure normal plant growth^20^. Typically, the accumulation of osmotic regulatory substances (proline, soluble sugars and soluble protein) is one of the basic characteristics of plants to adapt to drought stress^21^. For example, Shoukun Dong et al*.* found that the content of osmoregulatory substances such as proline, soluble sugars and soluble proteins increased sharply in soybean with increasing duration of drought stress^22^. Similarly, Yue-Bin Zhang et al*.* found that drought-resistant sugarcane varieties accumulated more soluble sugars, soluble proteins and proline than drought-sensitive sugarcane varieties under drought stress^23^. The *MDA* content is often measured as a suitable physiological index to reflect the degree of lipid peroxidation and stress tolerance in plants. The MDA content is often measured as a suitable physiological index to reflect the degree of lipid peroxidation and stress tolerance in plants^24^. In the present study, plant display some physiological and biochemical responses to inhibit the increase of MDA content under drought stress^25^. Zhou Q et al*.* reported that the MDA content of drought-sensitive soybean varieties increased significantly under drought stress, while the increase in MDA content of drought-resistant soybean varieties was relatively sluggish^26^. In our study, SOD, POD, proline, soluble protein and soluble sugar of ‘Guire 2’ tiller seedlings increased significantly with the increase of drought stress time and intensity, while MDA was maintained at the same level from the beginning to the end without significant changes. Apparently, ‘Guire 2’ varieties can rapidly initiate the defense strategy of reactive oxygen species scavenging and osmotic pressure regulation when subjected to drought stress, and eventually, through this defense strategy, mitigate the damage caused by drought stress.

It is not known at present which drought regulated genes are involved in activate the above anti-drought defense strategy in 'Guire 2' tiller seedlings. Thus, we identified gene modules and hub genes associated with the anti-drought defense strategy using the WGCNA following transcriptome analysis.

For the drakgreen module, LSG1-2 is an essential hub gene, which involved in ribosome biosynthesis and affects plant development^27^. Ribosomes are the centers of translation, which is the first step in the synthesis of many regulatory substances. Ribosomes biosynthesis has an impact on the synthesis of enzymes and various metabolic activities in plants. In Arabidopsis, AtLSG1-2 is essential for ribosome biosynthesis and ultimately affects growth hormone homeostasis and plant development^28^. In our project, the TCA cycle was significantly enriched in the darkgreen module, which is an important metabolic activity that promotes the synthesis of polysaccharides and amino acids, and has a wide range of regulatory mechanisms, including the regulation of oxidative stress and abiotic stress^29^. For example, Li et al^30^. found that drought-tolerant Sorghums responds to drought stress mainly by promoting the TCA cycle. Wang et al^31^. reported that drought-tolerant soybean varieties produce more antioxidant enzymes and osmotic substances, and TCA cycle was one of the core pathways of drought resistance in soybean varieties. In this study, the drakgreen module was positively correlated with the content of SOD, Pro, Source, and Portent. Thus, we speculate that the expression of LSG1-2 in "Guire 2" sugarcane may enhance ribosome biosynthesis, thus promoting the metabolic activities of the TCL cycle and eventually accumulating more antioxidant enzymes and osmoregulatory substances to defend against drought stress.

The spliceosome, a large ribonucleoprotein (RNP) complex, has the function of processing RNAs from a pre-RNA state to a mature mRNA thereby influencing RNA availability^32^. The pre-RNA is regulated by spliceosome to produce multiple mRNAs, which are further encoded into proteins, thereby enabling a limited number of genes to encode more proteins of different conformations, this process known as alternative splicing (AS)^33^. There is also increasing evidence that plants can encode more functional proteins in response to adversity stress through AS^34^. For example, drought stress induces crops such as maize^35^, soybean^36^, wheat ^37^and rice^38^ to undergo large-scale AS to form different function proteins,and thereby improve crop stress resistance. In our research, we had the same findings. The genes of darkgreen and grey60 modules were significantly enriched in the spliceosome pathway and both were positively correlated with SOD, Pro soluble sugars, and soluble proteins. Accordingly, it is postulated that spliceosome may affect the regulation of reactive oxygen species and osmotic pressure homeostasis when tiller seedlings of “Guire 2” in response to drought stress. Previous studies on sugarcane have documented similar findings. Jinlong Guo et al. reported that ScMYB2 forms two distinct transcript forms, ScMYB2S1 and ScMYB2S2, through AS under drought stress in sugarcane. ScMYB2S1 promotes the accumulation of MDA and proline, while ScMYB2S2 inhibits the elevation of MDA and proline^39^.

γ-aminobutyric acid (GABA) is a non-protein amino acid involved in various physiological processes. GABA is produced by a metabolic pathway known as the GABA shunt pathway, which tends to have a protective effect against drought stress in plants by increasing osmolytes regulation^40^. The skyblue module contains 1 hub gene annotated as GABA-T, which converts GABA to succinic semialdehyde (SSA). After a series of reactions, SSA forms succinate in the TCA cycle^41^. GABA shunt pathway increases plant drought tolerance through the accumulation of amino acids, organic acids and other osmotic compounds associated with secondary metabolism^42^.

Heat shock proteins (HSPs) are a set of chaperone proteins that function in the cytoprotection from denaturation or misfolding of cellular proteins corresponding to heat stress or other abiotic stress^43^. A Chinese study by Jianhua Xiang et al. found that OsHSP50.2 positively regulates drought stress tolerance in rice, probably through the modulation of reactive oxygen species (ROS) homeostasis^44^. A research about pepper line by Xiao-Hui Feng indicated that CaHsp25.9 confers drought stress tolerance to plants by reducing the accumulation of reactive oxygen species, enhancing the activity of antioxidant enzymes, and regulating the expression of stress-related genes^45^. Other stydies found that overexpression of an HSP70 gene which belongs to peony (*Paeonia lactiflora* Pall.) in Arabidopsis improved heat tolerance in transgenic Arabidopsis by ameliorating oxidative stress and maintaining cell membrane integrity^46^. Based on the results in previous research, we hypothesized that HSP positively regulates plant drought stress resistance by improving antioxidant capability, which is similar to our findings. In our WGCNA, HSP genes including Sspon.01G0038620-2C (HSP18.1), Sspon.04G0002960-1A (HSP24.1), Sspon.01G0038620-3D (HSP 18.1) and Sspon.03G0019770-1A (HSP16.1) were identified as grey60 module of hub genes, while grey60 was significantly and positively correlated with SOD. HSPs are divided into five families: HSP100s, HSP90s, HSP70s, HSP60s, and small heat shock proteins (sHSPs)^47^. In this study, the hub genes identified from the Grey60 module all belong to sHSPs. Present studies on plant sHSPs show that sHSPs enhance plant tolerance to heat and oxidative stresses mainly by increasing peroxidase activity ^48,49^. Although tiller seedlings of “Guire 2” in our study were subjected to drought stress rather than heat stress, they were also subjected to oxidative damage. Therefore, sHSPs may play a role in regulating SOD activity in the drought stress response of tiller seedlings. In addition, the upstream promoter regions of the *s*HSPs-encoding sequences usually contain multiple repeated 5′-nGAAnnTTCnnGAAn-3′ sequences (heat shock element, HSE), which are identified and bound by specific heat shock transcription factors (HSFs)^50^. Coincidentally, hub gene in the grey60 module also contains a heat stock transcription factor, HSFA6A. We speculate based on previous studies^51^ that HSFA6A may bind to HSE or promoters to initiate or substantially up-regulate HSPs genes.

As the drought stress level increased, significant changes happen at the transcriptional level in "Guire 2" tiller seedlings, out of which most were down-regulated. Most of down-regulated genes were mainly associated with photosynthesis, sugar metabolism and fatty acid synthesis, on these aspects, our results are in accordance with the previous studies^52,53^. Peiting Li et al. reported that photosynthesis-related processes showed significant enrichment between highly drought-resistant and weakly drought-resistant sugarcane under drought stress^54^. Xiaoning Cao et.al found that the highly enriched categories were related to starch and sucrose metabolism when drought-resistant and drought-sensitive millet variety were subjected to drought stress^55^. In our investigation, although the MDA values of tiller seedlings were maintained at a stable level during drought stress, seedlings growth and development were still affected by drought stress. The tiller seedling height and stem diameter of “Guire 2” significant reduce in the late stage of drought (day 15) compare with the CK group. WGCNA showed that greenyellow modules genes had a highly significant positive correlation with stem diameter traits. Interestingly, three of the hub genes identified from this module belong to the BHLH transcription factor, it is thus speculated that BHLH transcription factors may take a predominant regulatory role in the growth and development of tiller seedlings under drought stress. The majority of investigators consider that BHLH-type transcription factors are involved in plant growth, development and abiotic stress responses^56^. For example, Yufei Liang et al. reported that SlbHLH96 improves drought resistance in tomato by activating genes encoding antioxidant, ABA signaling and stress-related proteins^57^; Chunjuan Li et al. propose that AhbHLH112 is localized in the nucleus and the over expression of this gene improves the drought tolerance of transgenic plants both in seedling and adult stages; Beibei Liang et al. suggested that PtrbHLH66 from Trifoliate Orange acted as a positive regulator of plant drought resistance by regulating root growth and ROS scavenging^58^. It is well kown that plants respond to drought stress through both *ABA*-dependent and *ABA*-independent pathways^59^. Numerous studies have reported that bHLH transcription factors enhance drought tolerance in plants mainly through ABA-dependent pathways^60–62^. In our research, the three bHLH genes in the greenyellow module are all annotated to BHLH148 in Sorghum bicolor and may be paralogous genes. OsbHLH148 interacts with OsJAZ proteins in a jasmonate signaling pathway leading to drought tolerance in rice, the researcher further found thatthe expression levels of OsbHLH148 after treatment with MeJA and ABA in combination were higher than those with MeJA or ABA alone^63^. Such observations support the synergistic effect of MeJA and ABA on OsbHLH148 expression, leading to the stress tolerance of transgenic rice. Together with previous observations that drought stress stimulates the biosynthesis of MeJA and ABA in rice^64^. these results suggest that OsbHLH148 is involved in drought. Curiously, our current analysis about bHLH showed some discrepancies with those reported previously. The ABA﻿ content of drought treated sugarcanne was significantly higher than that of control sugarcane from day 5 to day 15, but no significant correlation between the changing trends of ABA and bHLH transcription factors. Therefore, whether bHLH transcription factor affects the growth and development of tiller seedlings in drought stress through the ABA pathway requires further study.

## Conclusions

Our present research demonstrated that drought stress induced huge changes in gene expression profilies of "Guire 2" sugarcane tiller seedlings. In particular, the expression of most genes related to cell wall development, photosynthesis, sugar metabolism, and fatty acid metabolism were inhibited during drought stress. After 15 days of drought stress (SWC ≈ 7%), the leaves of "Guire 2" tiller seedlings showed much less withering than those of the main stems.

Base on the physiological data, we found that accumulation of osmotic adjustment substances and enhancement of antioxidant enzyme activities are contribute to alleviate damage caused by drought stress in "Guire 2" tiller seedlings. Our WGCNA revealed that *LSG1-2, ERF1-2, SHKA, TIL, HSP18.1, HSP24.1, HSP16.1* and *HSFA6A* may play essential regulatory roles as hub genes in increasing SOD, Pro, soluble sugar or soluble protein contents, respectively.

## Methods

### Experimental materials and design

The sugarcane variety "Guire 2" used in this study was bred by the Guangxi Subtropical Crops Research Institute. The experiments were conducted in Germplasm Resources Base at Guangxi Subtropical Crops Research Institute in Nanning, Guangxi, China (108°20′E, 22°53′N) during the spring of 2022 year. The stems of “Guire 2”with healthy and disease-free were selected, and single bud stems with a length of approximately 9 cm were cultivated as experimental plants after soaking for 30 min in 0.1% carbendazim. Sugarcane stems were planted in plastic pots (22 cm upper diameter, 16 cm lower diameter, 20 cm height) with one drainage holes approximately 2 cm diameter at the bottom. The soil for the experiment was mixed with sand in the ratio 3:1 (soil:sand). Each plastic pots was filled with approximately 6 kg of mixed soil, and approximately 3 kg of compound fertilizer was applied as the base fertilizer. One single bud stem was planted in one plastic pots, then watered normally every 2 days after planting until the tiller stage. We planted a total of 100 plants, and this was in preparation for subsequent sugarcane tillering and drought stress experiments.

The drought stress experiment began when the tiller seedlings reached a height of about 1 cm. The experiment was divided into control and drought-stressed treatment groups. Drought stress was imposed during the tillering stage of "Guire 2" sugarcane by stopping irrigation, and the control group was irrigated every 2 days as normal. Soil water content, tiller seedlings height and stem diameter were measured every 2 days. Treatment and control tiller seedlings were collected on days 5, 9 and 15 at the beginning of the experiment, and marked as T1, T2, T3, CK1, CK2, and CK3, respectively. Three biological replicates were set for each group (each biological replicate contained five individuals). All fresh samples were stored at −80 °C until physiological assays and transcriptome sequencing.

### Determination of soil water content

Soil water content (SWC) was determined by weighing the soil before and after drying^65^. Soil (15 g) was taken at a uniform depth of about 15 cm from the surface. Fresh weight of the soil (three replicates from each treatment) was recorded. The dry weight was determined after the soil was dried in an oven at 80 °C for at least 24 h to a constant weight.

### Determination of height and stem diameter

The height and stem diameter of “Guire 2” tiller seedlings were recorded using vernier calipers. In particular, the middle part of the tiller seedlings was selected for the stem diameter measurement. Trait indicators were measured every two days.

### Determination of physiological changes

The actvites of anti-oxidative enzymes (SOD, POD) were examined using specific detection kits (Grace Biotechnology Co.,Ltd, Suzhou, China) with the help of microplate reader, according to the manufacturer’s instructions. The MDA content of tiller seedlings were measured by thiobarbituric (TBA) method as described by Hussain et al.^66^. Proline content was detected using the sulfosalicylic acid method according to Elasad et al*.*^67^. Soluble protein was assessed with coomassie brilliant blue G250 staining method^68^. The starch content was determined by an anthrone colorimetric method^69^. We measured soluble sugar contents by an enzymatc method using specific detection kits (Grace Biotechnology Co.,Ltd, Suzhou, China) ^70^.

The contents of endogenous hormones of “Guire 2” tiller seedlings were determined using the Enzyme-Linked Immunosorbent Assay (Elisa) technique. The method of extraction and determination of hormones, including GA, ABA, IAA, CTK and ZT, were measured as described by Wu et al. ^71^.

### RNA extraction, library construction and sequencing

The total RNA was extracted from tiller seedlings using Trizol reagent kit (Invitrogen, Carlsbad, CA, USA). RNA quality was assessed on an Agilent 2100 Bioanalyzer (Agilent Technologies, Palo Alto, CA, USA) and checked using RNase free agarose gel electrophoresis. After total RNA was extracted, eukaryotic mRNA was enriched by Oligo(dT) beads. Then the enriched mRNA was fragmented into short fragments using fragmentation buffer and reversly transcribed into cDNA by using NEB Next Ultra RNA Library Prep Kit for Illumina (New England Biolabs, Ipswich, MA, USA). The purified double-stranded cDNA fragment were end repaired, A base added, and ligated to Illumina sequencing adapters. The ligation reaction was purified with the AMPure XP Beads(1.0X). Ligated fragments were subjected to size selection by agarose gel electrophoresis and polymerase chain reaction (PCR) amplified. The resulting cDNA library was sequenced using Illumina Novaseq6000 by Gene Denovo Bitechnology Co. (Guangzhou, China).

Clean data were obtained by filtering the raw data, and then aligned with the sugarcane reference genome (*Saccharum spontaneum* AP85-441 genome, http://sugarcane.zhangjisenlab.cn/sgd/html/index.html, accessed on Aug. 2022) using HISAT2.2.4 ^72^. The mapped reads of each sample were assembled by using StringTie v1.3.1^73^ in a reference-based approach. Genes differential expression analysis was performed by DESeq2^74^ software between two groups, and the genes with the parameter of false discovery rate (FDR) below 0.05 and absolute fold change was greater than or equal to 2. The DEGs and genes in the modules correlated with physiological and biological traits were subjected to the enrichment analysis for Gene Ontology (GO; http://www.Geneontology.org/) and KEGG (Kyoto Encyclopedia of Genes and Genomes; https://www.kegg.jp/) pathways. Significant GO and KEGG pathways were identified with the criterion of qvalue < 0.05.

### Co-expression network construct and analysis

Co-expression networks were constructed using WGCNA(v 1.47) package in R^75^. After filtering genes with expression below 15, gene expression values were imported into WGCNA to construct co-expression modules using the automatic network construction function blockwiseModules with default settings, except that the power is 10 (Supplementary Fig. 3), TOMType is unsigned, mergeCutHeight is 0.25, minModuleSize is 50. And then, the Pearson correlation analysis was used to identify modules significantly (Pearson’s correlation coefficient >|0.7|) associated with physiological or biological traits of “Guire 2” tiller seedlings under drought stress. Final, the modules network was mapped using Cytoscape 3.7.2.

### Quantitative real-time PCR analysis

qRT-PCR analysis was performed to verify the expression of candidate hub genes. *GAPDH* gene is used as the internal reference gene. The qTOWERE2.2 (AnalytikJena, German) instrument were used for real-time fluorescence quantitative PCR (qRT-PCR). The 20 μL reaction volume contained 1 μL of diluted cDNA, 0.4 μL of forward and reverse primers (10 μM), 10 μL of 2 × Universal SYBR qPCR Master Mix (Vazyme, China) and 8.2 μL of ddH_2_O. The PCR amplification were performed with 95 °C for 3 min, followed by 40 cycles of 95 °C for 15 s, 58 °C for 15 s, then followed by 72 °C for 20 s. The relative quantification was calculated by 2^−ΔΔCT^ method. Three independent biological replicates were designed here.

### Statistical analysis

Statistical analysis was performed with GraphPad Prism 8.0.2. All experimental data were expressed as mean ± standard deviation (SD), and differences between treatments were analyzed using unpaired t-test. Differences between treatments were analyzed by one-way ANOVA test.

### Ethical approval

This article does not contain any studies with human participants or animals performed by any of the authors.

### The plant ethics statement

In this study, the plant material was "Guire 2" sugarcane variety. The cultivar was selected by Guangxi Subtropical Crops Institute, and passed the validation of Ministry of Agriculture and Rural Affairs of the People’s Republic of China in July 2020. Registration number: GPD Sugarcane (2020) 450040, Variety Name: “Guire 2”. We state that the research of “Guire 2” sugarcane comply with Guangxi Subtropical Crops Institute, China, and international legislation.

### Supplementary Information

Supplementary Table 1.

Supplementary Table 2.

Supplementary Table 3.

Supplementary Table 4.

Supplementary Table 5.

Supplementary Table 6.

Supplementary Table 7.

Supplementary Table 8.

Supplementary Figures.

## Data Availability

The sequencing data were submitted to NCBI SRA database with the accession number PRJNA955119.
