# Supplementary material for: Transcriptome and WGCNA reveal hub genes in sugarcane tiller seedlings in response to drought stress
Source: Sci Rep. 2023 Aug 7;13:12823. doi: 10.1038/s41598-023-40006-x (PMC10406934; doi:10.1038/s41598-023-40006-x)
Supplement: Supplementary file 9 — Supplementary Figures. [file 41598_2023_40006_MOESM9_ESM.doc]

**Transcriptome and WGCNA reveal hub genes in sugarcane tiller seedlings in response to drought stress**

Authors: Yuwei Tang, Jiahui Li, Qiqi Song, Qin Chen, Qinliang Tan, Quanguang Zhou, Zemei Nong &Ping Lv


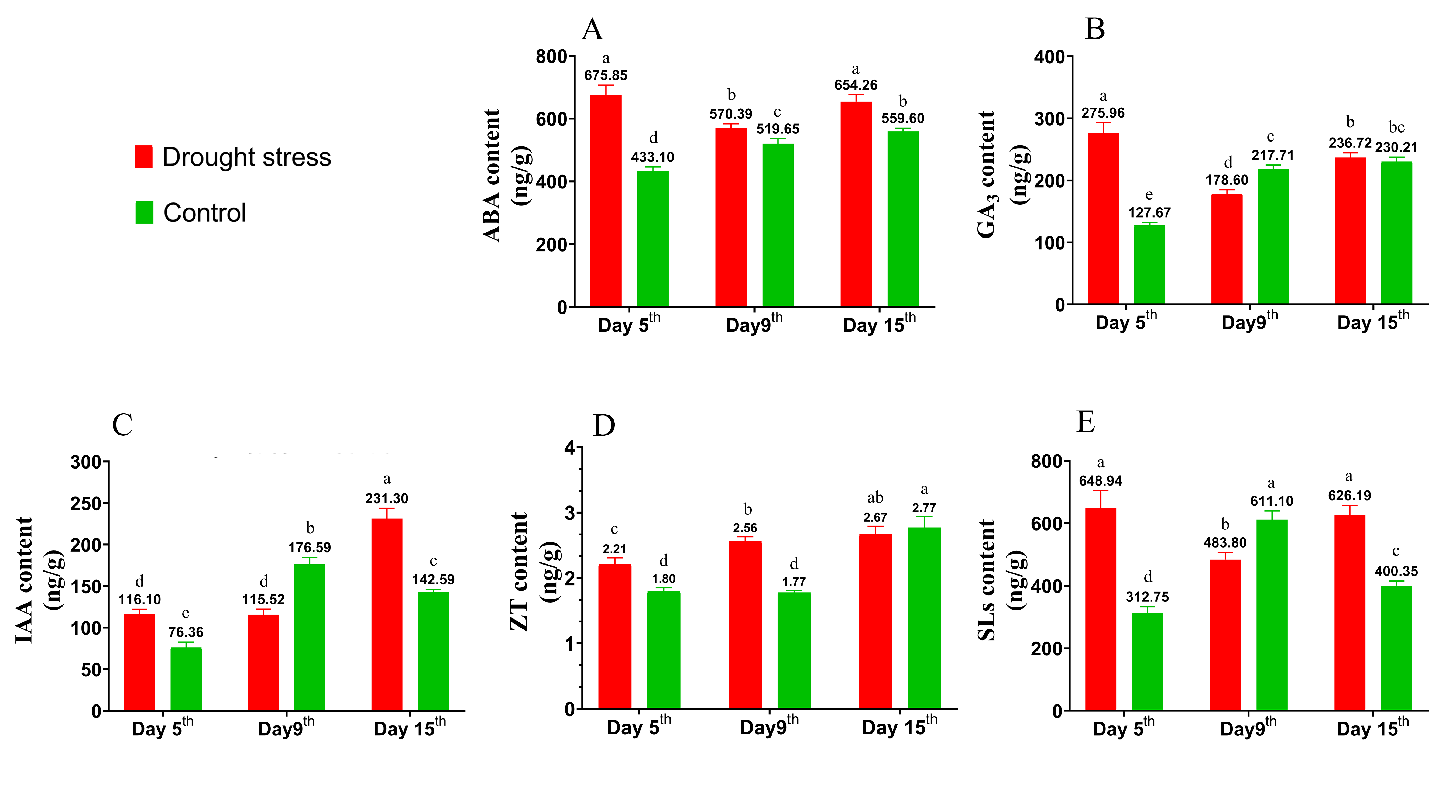


**Supplementary Figure 1.** Endogenous hormones changes of “Guire 2” tiller seedlings under drought stress (treated group) or well watering treatment (control group)**.** Different letters (a–c) represent significant differences (LSD, P < 0.05). **(A)** Abscisic Acid (ABA) content. **(B)** Gibberellin A_3_ (GA_3_) content. **(C)** Indoleacetic acid (IAA) content. **(D)** Zeatin (ZT) content. **(E)** Strigolactones (SLs) content.

**
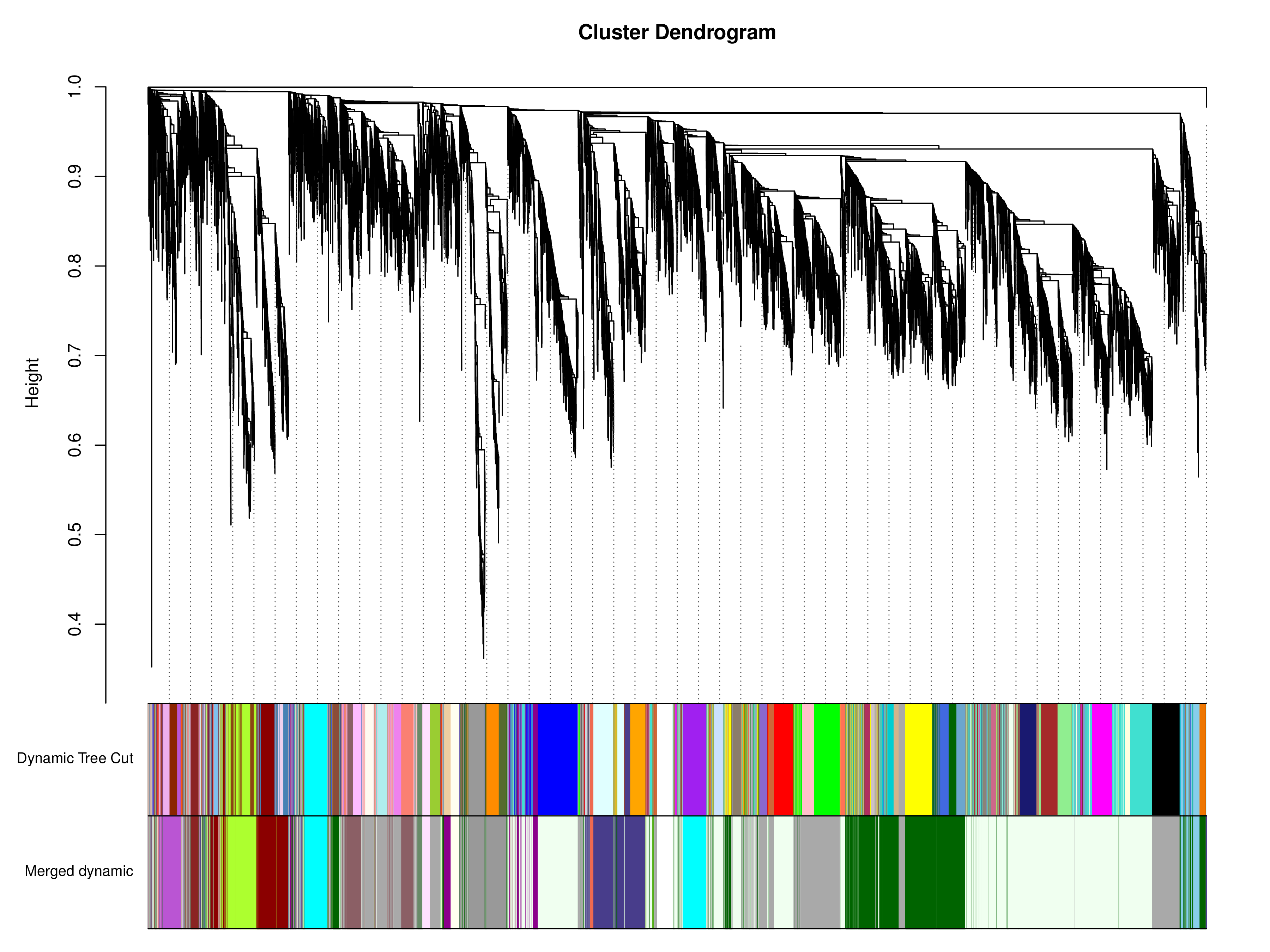
**

**Supplementary Figure 2** The clustering dendrogram of genes identifying the WGCNA modules.


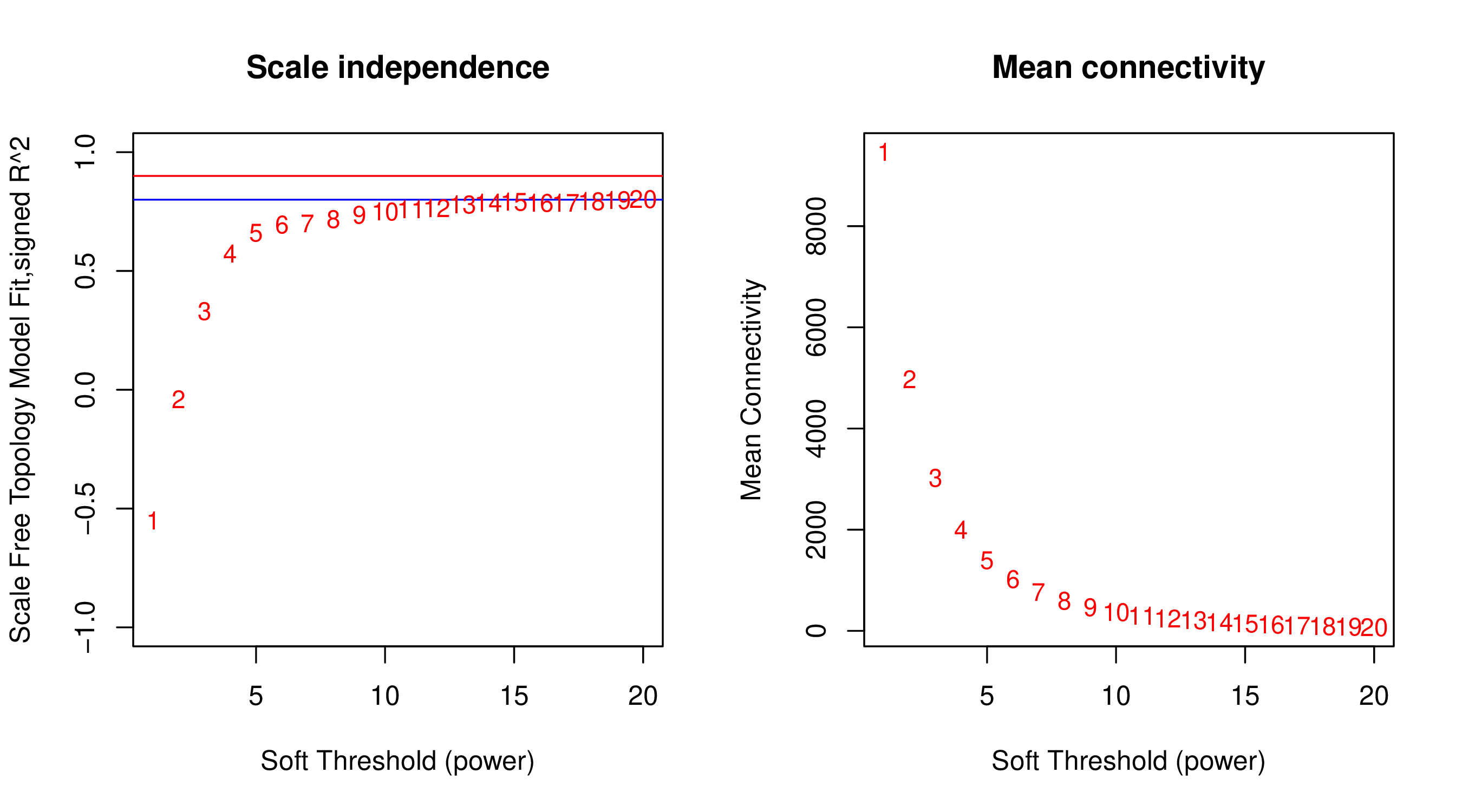


**Supplementary Figure 3** Graph of Power value. Left: the abscissa represents the power value, the ordinate represents the correlation coefficient, the blue line represents the correlation coefficient of 0.8, and the red line represents the correlation coefficient of 0.9. Right: the abscissa represents the power value, and the ordinate represents the average connectivity of genes.
